# The importance of peer reviewed, PubMed® listed journals in the age of Open Access journal publishing

**DOI:** 10.1093/jhps/hnaf031

**Published:** 2025-08-04

**Authors:** Richard E Field

**Affiliations:** City St George’s University of London

Anyone reading a paper published in an Open Access (OA) journal will be looking at a computer, tablet, or smartphone screen. They will have accessed the article *via* the Internet with no requirement to pay a subscription or fee. The OA model is increasingly popular and uptake has grown rapidly through the first two decades of this century [1, 2]. In 2011, a paper by Laakso *et al.* [3] reported that in the year 2000, around 195 000 OA articles were published in 740 OA journals and that in 2009, these numbers had grown to 191 850 articles in 4769 OA journals. In 2020, the number of OA journals had risen to 14 344 [4]. The Directory of Open Access Journals (DOAJ) currently lists 11 147 480 article records in 21 559 OA journals. Just over half (13 712) of the OA journals do not charge any fees to the authors or readers [5]. Approximately 25% of the journals enjoy *Index Medicus*® and MEDLINE® indexing on PubMed® [6]. Peer reviewed and PubMed listed OA journals, such as Journal of Hip Preservation Surgery (JHPS), are mostly funded by charging authors an Article Processing Charge (APC) [7]. Many academic institutions and research funders offer financial support for OA APCs through block grants, publisher agreements, and direct APC funding [6, 8–11].

Despite the global rise in OA journal publications, the globalization of trade has faltered since the 2019 COVID pandemic, the Ukrainian war, and the recent changes to international tariffs on imported goods by the US government. While OA journals have proven immune to these challenges, all areas of surgical practice will be affected by more restricted access to surgical equipment for the next few years. It should be anticipated that all surgical specialties will experience increasing pressure to demonstrate the efficacy and cost effectiveness of their interventions [12, 13]. Also, if barriers to international trade persist, global implant and equipment suppliers may become less able to compete with local suppliers and new providers may emerge to address local markets. If such change occurs, surgeons may find themselves reporting on interventions that are undertaken using equipment that is difficult to access in other geographic locations.

In this issue, we present a paper from Mathew Yuro and colleagues [14] analysing correlations between individual ‘international hip outcome tool 12’ (iHOT-12) items and overall iHOT-12 score within and across timepoints, to identify the subset of patients who will be best served by surgical treatment of femoroacetabular impingement. The article combines the outcomes of eight surgeons from eight medical centres. All had a minimum of 9 years of experience and undertook at least 150 hip arthroscopy surgeries per year. The study group comprised 3493 patients from whom three 170 iHOT-12 surveys were obtained preoperatively, 554 at 6-month, 797 at 1 year, 1114 at 2-year, and 532 at the 5-year postoperative timepoints. The manuscript is rich in statistical analysis and provides robust evidence that will guide surgeons who need to identify where limited healthcare resources may be best allocated.

On the theme of picking winners, Anil Ranawat’s team [15] has provided a lucid and valuable contribution in their paper looking at the time it takes to achieve the minimal clinically important difference after open proximal hamstring repair and which patients fare best. The article demonstrates that a history of prolonged pre-operative symptoms is a poor prognostic indicator for a successful outcome but that subjects with more severe preoperative symptoms are more likely to fare well with surgery.

In addition to picking winners, Hip Preservation surgeons need to know how their surgery will improve their patients’ mobility and ability to perform activities of daily living. A systematic review and meta-analysis looking at changes in physical impairments following arthroscopic surgery for femoroacetabular impingement syndrome has been provided by Charlotte Marshall and the team working with her in Melbourne, Australia [16]. The article focusses on hip movement and kinematics rather than the patient satisfaction metrics that are normally focussed upon by Hip Preservation surgeons [17–19]. The data provided in this article will be of particular value to therapists guiding patients through their post-operative rehabilitation; both to help the therapists set realistic rehabilitation goals and measure their patients’ progress.

In addition to the title specific information that these three papers provide, they also demonstrate that the Hip Preservation community understands the need to deliver value for money interventions and that this need will increase if global events make the allocation healthcare resources and access to equipment more challenging.

While the Internet enables OA journals to disseminate research findings, it is also the medium through which social media can be used to share ideas, techniques, and innovations. A reviewer of a manuscript, recently submitted to JHPS, observed that an innovative surgical technique described in the manuscript was one that the reviewer had never seen in clinical practice and was only aware of through LinkedIn. Securing objective and detailed reviews of manuscripts can be challenging and it is not uncommon that between 8 and 12 reviewers need to be approached to secure two helpful reviews. This delays decisions and is enormously frustrating for authors seeking to publish their research. We remain enormously grateful to members of the Hip Preservation community who give up their time to undertake manuscript reviews for the JHPS. Your thoughtful comments are invaluable to the peer review process, and we hope that these assignments help you better understand how to present your work when you submit manuscripts for publication. Your help is deeply appreciated and it is only through your continued support that the Hip Preservation community will maintain JHPS as the premier OA, peer reviewed, PubMed listed platform to present its work.

Conflict of interest: None declared.
